# Cross cultural translation, adaptation and reliability of the Malay version of the Canadian Acute Respiratory Illness and Flu Scale (CARIFS)

**DOI:** 10.1186/s12955-015-0336-z

**Published:** 2015-09-04

**Authors:** Anna Marie Nathan, Rafdzah Zaki, Rachael Rozario, Nurul Dhania, Siti Nur Sabrina Mohd Hamirudin, Kah Peng Eg, Sze Ying Kee, Cindy Teh, Kartini Abdul Jabar, Caroline Westerhout, Surendran Thavagnanam, Jessie de Bruyne

**Affiliations:** Department of Paediatrics, University Malaya, 50603 Kuala Lumpur, Malaysia; University Malaya Paediatric and Child Health Research Group, University Malaya, 50603 Kuala Lumpur, Malaysia; Department of Social & Preventive Medicine, Faculty of Medicine, Julius Centre University of Malaya, 50603 Kuala Lumpur, Malaysia; Department of Paediatrics, University Putra Malaysia, 43400 Serdang, Selangor Malaysia; Department of Microbiology, University Malaya, 50603 Kuala Lumpur, Malaysia; Department of Biomedical Imaging, University Malaya Medical Centre, 50603 Kuala Lumpur, Malaysia

## Abstract

**Background:**

The Canadian Acute Respiratory Illness and Flu Scale (CARIFS) is a parent-proxy questionnaire that assesses severity of acute respiratory infections in children. The aim was to (a) perform a cross-cultural adaptation and (b) prove that the Malay CARIFS is a reliable tool.

**Findings:**

The CARIFS underwent forward and backward translations as recommended by international guidelines. A pilot study was performed on the harmonised version and the final version of the Malay version of CARIFS was produced. A test-retest, 1 h apart, was then performed on parents with children less than 13 years old, admitted with a respiratory tract infection. Parents of children with asthma and who were not eloquent in Malay, were excluded. The data was analysed for consistency (Cronbach’s alpha) and reliability (test-retest co-efficient). Thirty-three parents were recruited. Children were aged median (IQR) 6 (2.8, 13.3) months with a male: female ratio of 22:11 and 88 % were Malays. Parents were interviewed at median (IQR) 6 (3, 11.5) days of admission. The Cronbach’s α coefficient was 0.70 for all items. The test–retest reliability analysis had a minimum and maximum intraclass correlation coefficient of 0.63 and 0.97 respectively. Clinically, the longer patients were admitted, the lower the severity score (*r* = −0.35, *p* < 0.05), indicating that they were getting better.

**Conclusion:**

The Malay version of CARIFS is a valid and reliable tool to determine severity of respiratory illness in children. Parent-centred questionnaires are useful and should be an adjunct to other methods, in monitoring response to treatment.

**Electronic supplementary material:**

The online version of this article (doi:10.1186/s12955-015-0336-z) contains supplementary material, which is available to authorized users.

## Introduction

Acute respiratory infections (ARI) account for the majority of childhood illnesses [1] comprising 30–50 % of health facility visits and 20–40 % of hospital admissions [2].

Acute respiratory scoring systems are vital in assessing severity of illness and response to treatment. However, questionnaires for use in paediatric respiratory disease, besides asthma, are scarce. Although the Wang and the Kristjansson Respiratory Scores have been validated for bronchiolitis [3, 4], they require medical expertise and thus are more tedious to apply in both the clinical and research settings. Inter-observer reliability is an important consideration, when choosing a questionnaire with different personnel assessing the child at different time points of illness.

The Canadian Acute Respiratory Illness and Flu Scale (CARIFS) was designed to assess the severity of acute respiratory infections in children [5]. It is a parent-proxy questionnaire measuring 3 conceptual dimensions of illness: symptoms/physiological, functional compromise and impact of illness/burden on parents. As it precludes the need for medical expertise, it is easily administered and, being completed by the same person, it reduces the problems of inter-observer reliability. It has been shown to be a valid tool in English and Ukrainian [6–11].

The aim of this study was to (a) perform a cross-cultural adaptation of the CARIFS for use in Malaysia and (b) demonstrate that the Malay version of CARIFS is a reliable tool.

## Methodology

### Translation process

In this study, we followed the linguistic validation process undertaken by the PedsQL [12]. Permission from the author of the original paper [5], Dr Benjamin Jacobs, was obtained to both translate and use the translated questionnaire.

The CARIFS comprises 18 questions, answered on a 4-point ordinal scale (no problem = 0, minor problem = 1, moderate problem = 2, major problem = 3) with attention to 3 domains: symptoms (Q4,10-16), function (Q1,3,5,6,17) and parental impact (Q2,7-9,18) (Additional file 1). A higher score indicates more severe illness. As certain items may not be applicable to young children e.g. headache (Q10), sore throat (Q11) and muscle aches/pains (Q12), these could be marked as don’t know or not applicable, with no score allotted to them. The final total mean score = (total score/number of items answered) x 18. [5] The CARIFS is done twice in a day, about 6–8 h apart, to document change in clinical severity.

**Fig. 1:**
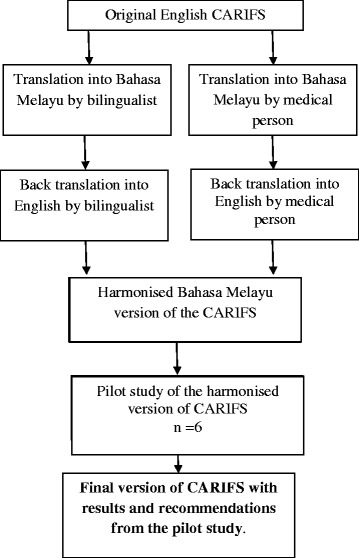
Method of translation of the Canadian Acute Respiratory Illness and Flu Scale (CARIFS) from English to Malay

The English version of the CARIFS underwent 2 forward and 2 backward translations by native language speakers (medical and non-medical), bilingual in both English and Malay, as shown in Fig. 1. Translations were tabulated and a round table discussion was held to reconcile issues with certain words, especially concerning cultural impact, an important aspect when adapting a questionnaire (Additional file 2). Six randomly selected parents of patients admitted with acute respiratory infections pilot tested the final harmonised version. Results are shown in Additional file 3. The final version of the questionnaire was then used in the test-re-test population (Additional file 4).

### Study design and patient selection for the test-retest

This was a cross sectional study, from 26^th^ August till 14^th^ October 2014. Local ethics committee (University Malaya Medical Centre Ethics Committee) approval was obtained and parents gave written consent before enrolment.

A minimum sample size of 19 was required to achieve 80 % power at alpha level of 5 % with expected correlation coefficient of 0.6. Sample size was increased to 38.

Parents were recruited via convenience sampling. Parents of children aged 12 years and less, admitted with a diagnosis of pneumonia or bronchiolitis were included. Parents unable to read Malay or whose children had asthma (or >2 wheezing episodes), were severely ill or had significant mental disabilities were excluded.

Demographic information was collected: age, sex, diagnosis, co-morbidity, symptoms at presentation, relationship of caregiver, day of illness and duration of hospitalization.

### Statistical analyses

Data was analysed using Statistical Package for Solutions and Services (SPSS) version 18.0. Demographic statistics were used to analyse the demographic characteristics. Mean (standard deviation [SD]) was used to describe normally distributed continuous variables and median (interquartile range [IQR]) for data that was not normally distributed.

For the validation of the questionnaire, three important aspects of reliability were considered: internal consistency, inter-rater reliability and test re-test reliability.

Cronbach’s alpha was used to assess the internal consistency reliability. When the true score was not measured at all and there was only an error component, alpha equaled zero. Alpha equaled one when all items measured only the true score and there was no error component. In this study Cronbach’s alpha value of ≥ 0.60 was considered an acceptable value.

Test-retest reliability measured stability of a result over time. This was done by administering the same test to the same subject at two points in time. The CARIFS was self-administered twice, one hour apart. Parents were reminded to recall how their child was on first testing when they completed the second test. Intraclass Correlation Coefficient (ICC) was used to measure inter-rater reliability in this study. ICC may be conceptualized as the ratio of between-groups variance to total variance. ICC is equal to one only when there is no variance due to the raters and no residual variance to explain. ICC of > 0.6 was considered as acceptable in this study.

Inter-rater reliability measures homogeneity, and to establish the extent of consensus on use of the instrument by those who administer it. Cohen’s Kappa for inter-rater reliability was used to assess inter-rater reliability in this study. Kappa value of < 0.40 indicates poor to fair agreement, 0.41–0.60 moderate agreement, 0.61–0.80 good agreement and >0.80 excellent agreement [1] Finally, to assess the ability of the questionnaire to detect severity of illness, bivariate correlation (Pearson’s correlation coefficient) was used to determine if there were significant correlations between the total score, different domains of the score and duration of hospitalization from admission till the day of interview.

## Results

Translation of the questionnaire was done successfully (Additional file 2). During the pilot study, linguistic issues with 3 questions were discovered: “Clinginess”, “Not interested in what’s going on and “Unable to get out of bed” (Additional file 3). While identified by parents as difficult to understand, yet all parents were able to explain these questions correctly. This initial difficulty may be due to the rare local usage of these words to describe their children. Finally, questions like “headache”, “muscle pain” and “sore throat” were identified as questions not applicable to young, non-verbal child.

Thirty-three parents were recruited for the test-retest. The median (IQR) age of the children was 6.0 (2.8, 13.3) months. The male: female ratio was 22:11. Eighty-eight percent of the participants were Malay, 6.0 % Chinese and 6.0 % Indians. Twenty patients had pneumonia and thirteen bronchiolitis. Six had other comorbid conditions. The parents were interviewed at median (IQR) of 6 (3, 11.5) days of admission. Mostly mothers (88 %), answered the questionnaires. The test-retest time interval between the first and second interview was median (IQR) 1.03(1.00, 1.10) hrs. Many parents found the following questions “not applicable” to their child: “headache” (51.5 %), “sore throat” (36.4 %), “cannot get up in the morning” (42.0 %), “muscle ache” (54.5 %), “don’t feel like doing anything” (24.0 %).

Only 3 % had a ceiling effect (52–54) with nobody at the floor (score 0–2). The Cronbach’s α coefficient was 0.70 for all items and 0.72, 0.84, and 0.77 for each domain (symptom, function and parental impact) respectively. The test–retest reliability analysis showed good result for all items in the questionnaire, with minimum ICC of 0.63 for Question 4 and maximum ICC of 0.97 for Question 18 (Table 1). Results for inter rater reliability are also shown in Table 1. The total mean (standard deviation) score [(total score/ number of questions answered) x18] for the first and the second measurement was 21.28(12.95) and 19.22(14.33) respectively. There was a significant correlation between the different domains of the questionnaire: symptoms with function (*p* < 0.001), symptoms with impact on child (*p* < 0.001) and function with impact (*p* < 0.001). There was weak negative association between the CARIFS score at first interview and the duration of hospitalization interview (*r* = −0.35, *p* < 0.05) indicating that the lower the severity score at the time of interview, the longer the child had been in hospital before the interview. The negative associations were seen in all three domains with the duration of hospitalisation before interview; symptom *r* = −0.281(*p* = 0.11), function *r* = −0.36 (*p* = 0.04), and parental impact *r* = −0.37 (0.04). When we analysed this clinical severity in children without co-morbidities, there was a greater negative association between the total mean score with duration of hospitalization (*r* = −0.42, *p* = 0.03). Similarly this was seen between the duration of hospitalization and the different domains: symptoms *r* = −0.32 (*p* = 0.11), function *r* = −0.44(*p* = 0.02) and parental impact *r* = −0.36(*p* = 0.07) with a significant association between function and duration of hospitalization.

**Table 1:** Results of the questionnaire validation

Internal consistency		Test re-test reliability (consistency)		Inter rater reliability (agreement)	
All items *N* = 18 Cronbach’s alpha = 0.703	Cronbach’s alpha if item deleted	ICC	*p*-value	Kappa	*p*-value
Q1	0.692	0.805	<0.001	0.551	<0.001
Q2	0.697	0.651	<0.001	0.538	<0.001
Q3	0.687	0.775	<0.001	0.517	<0.001
Q4	0.682	0.628	<0.001	0.342	0.001
Q5	0.679	0.855	<0.001	0.590	<0.001
Q6	0.678	0.756	<0.001	0.528	<0.001
Q7	0.684	0.724	<0.001	0.530	<0.001
Q8	0.698	0.655	<0.001	0.464	<0.001
Q9	0.680	0.794	<0.001	0.517	<0.001
Q10	0.691	0.885	<0.001	0.643	0.001
Q11	0.688	0.816	<0.001	0.466	<0.001
Q12	0.691	0.649	0.04	0.632	0.011
Q13	0.679	0.846	<0.001	0.466	<0.001
Q14	0.688	0.651	<0.001	0.447	<0.001
Q15	0.682	0.806	<0.001	0.463	<0.001
Q16	0.713	0.729	<0.001	0.467	<0.001
Q17	0.686	0.887	<0.001	0.600	<0.001
Q18	0.684	0.970	<0.001	0.808	<0.001

## Discussion

The translated Malay version of the CARIFS was consistent and reliable in assessing severity of paediatric respiratory infections.

This questionnaire has undergone rigorous translation with great effort during the harmonization to ensure cultural adaptation while preserving semantic equivalence.

The pilot study was successful as parents could relate to all the questions except for “headache”, “sore throat”, “muscle aches”, “cannot get up in the morning” and “don’t feel like doing anything”, due to the young age of the patients. In some studies, the first three are removed as they are inappropriate for young children [11].

The psychometric properties of the CARIFS were satisfactory and strong support for its validity was demonstrated by the high Cronbach’s alpha. The reliability of the CARIFS was established for both internal and test-retest consistency,

The CARIFS is similar to the Wisconsin Upper Respiratory Symptom Survey (WURSS21), except that it is for use in children. The domains in the WURSS21 are similar to the CARIFS, except that the CARIFS also looks at the burden of illness on parents [13]. Shepperd S et al., in 2004, utilised the CARIFS in 178 young children with ARI, in an outpatient setting. Parents found the questionnaire easy to use. It was a valid tool in assessing functional disability and burden of illness on the family but not physiological severity as it did not correlate with physician’s assessment of severity [10]. The authors explained this discrepancy could be due to the “insensitivity” of the physician-completed visual analog scale. The other problem could also be the lack of consistency between what parents interpret as a “problem” as it is dependent on various other factors like parental anxiety level and previous experiences with illnesses. In our study, the Cronbach’s alpha was also marginally lower in the physiological or symptoms scores.

To investigate if the questionnaire was sensitive in gauging severity of illness, we correlated the duration of hospitalization before interview, assuming that the longer the treatment in hospital, the greater the improvement compared to if the child was just admitted, whereby the severity of illness would be greater. We found that the lower severity scores were seen in children who had been admitted longer. This correlation was more significant when we removed patients with co-morbities, as duration of hospitalisation may be affected by problems other than an acute respiratory problem. Although this may seem contrary to common perception, however in normal, uncomplicated cases, patients would improve as they stayed longer in hospital, as opposed to if the interview was done very shortly after admission, which would be the peak of symptoms and illness. In both the analyses, the most significant association was seen with perceived function of the child and the total mean score. This is interesting as clinically, the first observed improvement in any sick child would be the activity of the child rather than the symptoms, which may persist. This has previously been noted in other studies, that this tool is not good at detecting physiological severity but better at detecting functional disability and burden of illness on the caregiver [10].

Limitations of this study are recognised. Number of parents recruited for the test-retest could have been increased. We did not use clinical parameters other than duration of hospitalisation, as a marker of sensitivity of the questionnaire in detecting severity of illness. This is not a validation study and that is a future priority. As this is a hospital based study in only one hospital, this result may not be representative of the general population.

In conclusion, the CARIFS has been successfully translated and cross-culturally adapted into Malay, to produce a valid and reliable tool, determining severity as well as progression of respiratory illness. Parent-centered questionnaires are useful and should be an adjunct to other methods in monitoring response to treatment.

## Additional files

Additional file 1:
**English version of the Canadian Acute Respiratory Illness and Flu Scale (CARIFS).** (DOC 43 kb) Additional file 2:
**Translations of CARIF FLU and final harmonised version.** (DOC 100 kb) Additional file 3:
**Pilot testing review report.** (DOC 98 kb) Additional file 4:
**Canadian Acute Respiratory Illness and Flu Scale (CARIF).** (DOCX 16 kb)

## References

[1] Carabin H, Gyorkos TW, Soto JC, Penrod J, Joseph L, Collet JP (1999). Estimation of direct and indirect costs because of common infections in toddlers attending day care centers. Pediatrics.

[2] Vashishtha VM (2010). Current status of tuberculosis and acute respiratory infections in India: much more needs to be done!. Indian Pediatr.

[3] Wang EEL, Milner RA, Navas L, Maj H (1992). Observer agreement for respiratory signs and oximetry in infants hospitalized with lower respiratory infections. Am Rev Respir Dis.

[4] Chin HJ, Seng QB (2004). Reliability and validity of the respiratory score in the assessment of acute bronchiolitis. The Malaysian Journal of Medical Sciences: MJMS.

[5] Jacobs B, Young NL, Dick PT, Ipp MM, Dutkowski R, Davies HD, Langley JM, Greenberg S, Stephens D, Wang EE (2000). Canadian Acute Respiratory Illness and Flu Scale (CARIFS): development of a valid measure for childhood respiratory infections. J Clin Epidemiol.

[6] Gerasimov SV, Belova HA, Pavuk HL, Seniuk IM, Strekalina YI (2014). The Ukrainian version of the pediatric Canadian acute respiratory illness and flu scale: a linguistic validation study. Patient Relat Outcome Meas.

[7] Fischer JB, Prasad PA, Coffin SE, Alpern ER, Mistry RD (2014). Canadian Acute Respiratory Illness and Flu Scale (CARIFS) for clinical detection of influenza in children. Clin Pediatr (Phila).

[8] Shepperd S, Perera R, Bates S, Jenkinson C, Hood K, Harnden A, Mant D (2004). A children’s acute respiratory illness scale (CARIFS) predicted functional severity and family burden. J Clin Epidemiol.

[9] Whitley RJ, Hayden FG, Reisinger KS, Young N, Dutkowski R, Ipe D, Mills RG, Ward P (2001). Oral oseltamivir treatment of influenza in children. Pediatr Infect Dis J.

[10] Saunders NR, Tennis O, Jacobson S, Gans M, Dick PT (2003). Parents’ responses to symptoms of respiratory tract infection in their children. CMAJ.

[11] Harnden A, Perera R, Brueggemann AB, Mayon-White R, Crook DW, Thomson A, Mant D (2007). Respiratory infections for which general practitioners consider prescribing an antibiotic: a prospective study. Arch Dis Child.

[12] PedsQL Linguistic validation of the PedsQL-a Quality of Life Questionnaire. www.pedsql.org/translations.html

[13] Barrett B, Brown RL, Mundt MP, Thomas GR, Barlow SK, Highstrom AD, Bahrainian M (2009). Validation of a short form Wisconsin Upper Respiratory Symptom Survey (WURSS-21). Health Qual Life Outcomes.

