# Supplementary material for: Cross cultural translation, adaptation and reliability of the Malay version of the Canadian Acute Respiratory Illness and Flu Scale (CARIFS)
Source: Health Qual Life Outcomes. 2015 Sep 4;13:139. doi: 10.1186/s12955-015-0336-z (PMC4559942; doi:10.1186/s12955-015-0336-z)
Supplement: Additional file 1: — English version of the Canadian Acute Respiratory Illness and Flu Scale (CARIFS). (DOC 43 kb) [file 12955_2015_336_MOESM1_ESM.doc]

**Additional file 1: English version of the Canadian Acute Respiratory Illness and Flu Scale (CARIFS)**

|  | No problem | Minor problem | Moderate problem | Major problem | Don’t know or Not Applicable |
| --- | --- | --- | --- | --- | --- |
| 1.Poor appetite |  |  |  |  |  |
| 2. Not sleeping well |  |  |  |  |  |
| 3. Irritable, cranky, fussy |  |  |  |  |  |
| 4. Feels unwell |  |  |  |  |  |
| 5. Low energy, tired |  |  |  |  |  |
| 6. Not playing well |  |  |  |  |  |
| 7.Crying more than usual |  |  |  |  |  |
| 9.Clinginess |  |  |  |  |  |
| 10. Headache |  |  |  |  |  |
| 11.Sore throat |  |  |  |  |  |
| 12. Muscle aches or pains |  |  |  |  |  |
| 13.Fever |  |  |  |  |  |
| 14. Cough |  |  |  |  |  |
| 15.Nasal congestion, runny nose |  |  |  |  |  |
| 16.Vomiting |  |  |  |  |  |
| 17.Not interested in what’s going on |  |  |  |  |  |
| 18.Unable to get out of bed |  |  |  |  |  |

**Please mark on this line how sick your child is today:**

**Best possible health**

**Worst possible health**
