# Supplementary material for: Cross cultural translation, adaptation and reliability of the Malay version of the Canadian Acute Respiratory Illness and Flu Scale (CARIFS)
Source: Health Qual Life Outcomes. 2015 Sep 4;13:139. doi: 10.1186/s12955-015-0336-z (PMC4559942; doi:10.1186/s12955-015-0336-z)
Supplement: Additional file 2: — Translations of CARIF FLU and final harmonised version. (DOC 100 kb) [file 12955_2015_336_MOESM2_ESM.doc]

|  | **Original English Version** | **Medical malay translation** | **Medical back translation** | **Non-medical malay translation** | **Non-medical back translation** | **Final malay**  **version** |
| --- | --- | --- | --- | --- | --- | --- |
|  | **Additional file 2 Translations of CARIF FLU and final harmonised version**  ***Condition of patient:***  *No problem*  *Minor problem*  *Moderate problem*  *Major problem*  *Don’t Know / Not Applicable* | ***Keadaan pesakit:***  *Tiada masalah*  *Masalah kecil*  *Masalah yang*  *Agak serius*  *Masalah serius*  *Tidak diketahui/Tidak berkenaan* | ***Patient’s condition:***  *No problems*  *Small problem*  *Quite serious problem*  *Serious problem*  *Don’t know* | ***Keadaan pesakit:***    *Tiada masalah*  *Masalah kecil*  *Masalah sederhana*  *Masalah besar*  *Tidak tahu / tidak berkenaan* | ***Condition of patient:***  *No problem*  *Minor problem*  *Moderate problem*  *Major problem*  *Unknown / Not applicable* | ***Keadaan pesakit:***  *Tiada masalah*  *Masalah kecil*  *Masalah sederhana*  *Masalah besar*  *Tidak tahu / tidak berkenaan* |
| 1 | Poor appetite | Kurang selera makan | Reduced appetite | Kurangseleramakan | Poor appetite | Kurangseleramakan |
| 2 | Not sleeping well | Tidak nyenyak tidur | Difficulty sleeping | Tidaktidurnyenyak | Lack of sleep | Tidaktidurnyenyak |
| 3 | Irritable, cranky, fussy | Asyik menangis,mudah marah, tidak selesa | Irritable | Cepatmarah,  sukameragam dancerewet | Easily angered / mood swings / fussy | Asyik menangis,cepat marah, tidak selesa |
| 4 | Feels unwell | Rasa tidak sihat | Unwell | Tidaksedapbadan | Uneasy feeling | Rasa tidak sihat |
| 5 | Low energy, tired | Kurang bertenaga, penat | Lethargy | Kurangtenaga, letih | Lethargic | Kurang bertenaga,letih |
| 6 | Not playing well* | Tidakbermaindenganbaik | Less play | Tidakbermaindenganbaik | Lack of active participation | Tidakbermainsepertibiasa |
| 7 | Crying more  than usual | Menangislebihdaripadabiasa | Cries more | Menangislebihdaripadabiasa | Unusualfrequency of crying | Menangislebihdaripadabiasa |
| 8 | Needing extra care | Memerlukanlebihpenjagaan | Needs more attention | Perlupenjagaan yang lebih | Needing extra care | Perlulebihpenjagaan |
| 9 | Clinginess* | Sentiasaperludidukung/ di pegang | Wants to be constantly carried or held (Wants to be constantly consoled) | Tahapberdampingan | Level of affection | Berpaut |
| 10 | Headache | Sakitkepala | Headache | Sakitkepala | Headache | Sakitkepala |
| 11 | Sore throat | Sakittekak | Sore throat | Sakittekak | Sore throat | Sakittekak |
| 12 | Muscles aches  or pains | Ototlenguhatausakit | Muscle aches | Sengalatau  sakitotot | Muscle pain | Sakitotot |
| 13 | Fever | Demam | Fever | Demam | Fever | Demam |
| 14 | Cough | Batuk | Cough | Batuk | Cough | Batuk |
| 15 | Nasal congestion, runny nose | Hidungtersumbat, selsema | Runny nose | Hidungtersumbat, hingusmeleleh | Sinus / blocked / runny nose | Hidungtersumbat, hingusmeleleh |
| 16 | Vomiting | Muntah | Vomiting | Muntah | Vomiting | Muntah |
| 17 | Not interested in what’s going on* | Tidakberminatdenganapa yang sedangberlaku | Listless | Tidakberminatdenganapa yang sedangberlaku | Uninterested in surrounding  activities | Tidakberminatdenganapa yang berlaku |
| 18 | Unable to get  out of bed* | Tidakberdayauntukbangundarikatil | Not able to get out of bed | Tidakbolehbangundaripadakatil | Unable to wake up from bed | Tidakberdayabangundarikatil |

|  | ***Please mark on this line how sick your child is today:***  Very healthy    Least healthy  (Canadian Acute Respiratory Infection and ‘Flu Scale’) | ***Silatandakan di atasgarisantahapkesihatananakanda:***  Amatsihat  Paling tidaksihat  (Skala ‘Canadian Acute Respiratory Infection and ‘Flu’) |  | ***Silatandapadagarisaninitahapkesihatananakandahariini:***  Paling sihat  Paling kurang  sihat  (JangkitanPernafasanAkutdi Kanadadan  ‘SkalaSelsema’) | ***Please mark on the line the health level of your child today:***  Very healthy    Least healthy  (Canadian Acute Respiratory Infection and ‘Flu Scale’) | ***Silatandapadagarisaninitahapkesihatananakandahariini:***  Paling sihat  Paling tidak  sihat  (JangkitanPernafasanAkutdi Kanadadan  ‘SkalaSelsema’) |
| --- | --- | --- | --- | --- | --- | --- |

*words which were deemed “difficult to understand” by parents
