# Supplementary material for: Cross cultural translation, adaptation and reliability of the Malay version of the Canadian Acute Respiratory Illness and Flu Scale (CARIFS)
Source: Health Qual Life Outcomes. 2015 Sep 4;13:139. doi: 10.1186/s12955-015-0336-z (PMC4559942; doi:10.1186/s12955-015-0336-z)
Supplement: Additional file 4: — Canadian Acute Respiratory Illness and Flu Scale (CARIF). (DOCX 16 kb) [file 12955_2015_336_MOESM4_ESM.docx]

**Additional file 4 Canadian Acute Respiratory Illness and Flu Scale (CARIF)**

**Tarikh : _____________ Masa : ______________**

**Sila tanda di mana yang berkenaan untuk menilai tahap selsema dan batuk anak anda.**

|  | Tiada masalah | Masalah kecil | Masalah sederhana | Masalah besar | Tidak tahu / tidak  berkenaan |
| --- | --- | --- | --- | --- | --- |
| 1. Kurang selera makan |  |  |  |  |  |
| 2. Tidur tidak nyenyak |  |  |  |  |  |
| 3. Asyik menangis, tidak selesa |  |  |  |  |  |
| 4. Rasa tidak sihat |  |  |  |  |  |
| 5. Kurang bertenaga, letih |  |  |  |  |  |
| 6. Tidak bermain seperti biasa |  |  |  |  |  |
| 7. Menangis lebih kerap |  |  |  |  |  |
| 8. Perlu lebih penjagaan |  |  |  |  |  |
| 9. Berpaut / berdampingan lebih daripada biasa |  |  |  |  |  |
| 10. Sakit kepala |  |  |  |  |  |
| 11. Sakit tekak |  |  |  |  |  |
| 12. Sakit otot |  |  |  |  |  |
| 13. Demam |  |  |  |  |  |
| 14. Batuk |  |  |  |  |  |
| 15. Selsema |  |  |  |  |  |
| 16. Muntah |  |  |  |  |  |
| 17. Tidak berminat dengan  apa yang berlaku |  |  |  |  |  |
| 18. Tidak berupaya bangun dari katil |  |  |  |  |  |

**Sila tanda pada garisan ini tahap kesihatan anak anda hari ini :**

Paling sihat |--------------------------------------------------| Paling tidak sihat
