# Supplementary material for: Cross cultural translation, adaptation and reliability of the Malay version of the Canadian Acute Respiratory Illness and Flu Scale (CARIFS)
Source: Health Qual Life Outcomes. 2015 Sep 4;13:139. doi: 10.1186/s12955-015-0336-z (PMC4559942; doi:10.1186/s12955-015-0336-z)
Supplement: Additional file 3: — Pilot testing review report. (DOC 98 kb) [file 12955_2015_336_MOESM3_ESM.doc]

**Additional file 3 Pilot testing review report**

Parent respondent demographics

| Attribute | P1 | P2 | P3 | P4 | P5 | P6 |
| --- | --- | --- | --- | --- | --- | --- |
| Age ( years) | 31 | 31 | 28 | 26 | 32 | 27 |
| Race | Malay | Malay | Malay | Malay | Malay | Malay |
| Written Malay | Good | Good | Good | Good | Good | Good |
| Malay comprehension | Medium | Good | Good | Good | Good | Good |
| Education level | SPM | Degree | Degree | Diploma | Degree | Diploma |

Responses from parents on difficulty of questionnaire

1 (sangat sukar), 2 (sukar), 3 (sederhana), 4 (mudah) dan 5 (sangat mudah)

| Questions | P1 | P2 | P3 | P4 | P5 | P6 |
| --- | --- | --- | --- | --- | --- | --- |
| Q1 | 4 | 4 | 4 | 5 | 2 | 5 |
| Q2 | 4 | 4 | 4 | 5 | 4 | 5 |
| Q3 | 3 | 4 | 4 | 5 | 4 | 5 |
| Q4 | 3 | 4 | 4 | 5 | 4 | 5 |
| Q5 | 4 | 4 | 4 | 5 | 4 | 5 |
| Q6 | 4 | 4 | 4 | 5 | 3 | 5 |
| Q7 | 3 | 4 | 4 | 5 | 3 | 5 |
| Q8 | 4 | 4 | 4 | 5 | 3 | 5 |
| Q9 | 4 | 4 | 4 | 3 | 2 | 2 |
| Q10 | 2 | 2 | 2 | 5 | 4 | 5 |
| Q11 | 4 | 2 | 2 | 5 | 4 | 5 |
| Q12 | 2 | 2 | 2 | 2 | 4 | 5 |
| Q13 | 4 | 4 | 4 | 5 | 4 | 4 |
| Q14 | 4 | 4 | 4 | 5 | 4 | 4 |
| Q15 | 4 | 4 | 4 | 5 | 4 | 3 |
| Q16 | 4 | 4 | 4 | 5 | 4 | 5 |
| Q17 | 4 | 4 | 4 | 5 | 3 | 4 |
| Q18 | 3 | 4 | 4 | 5 | 4 | 5 |

Parent response on comprehension of the questionnaire: Faham (F), Tidak faham (TF)

| Questions | P1 | P2 | P3 | P4 | P5 | P6 |
| --- | --- | --- | --- | --- | --- | --- |
| Q1 | F | F | F | F | TF | F |
| Q2 | F | F | F | F | F | F |
| Q3 | F | F | F | F | F | F |
| Q4 | F | F | F | F | F | F |
| Q5 | F | F | F | F | F | F |
| Q6 | F | F | F | F | TF | F |
| Q7 | F | F | F | F | F | F |
| Q8 | F | F | F | F | F | F |
| Q9 | F | F | F | F | TF | TF |
| Q10 | TF | TF | TF | F | F | F |
| Q11 | F | TF | TF | F | F | F |
| Q12 | TF | TF | TF | TF | F | F |
| Q13 | F | F | F | F | F | F |
| Q14 | F | F | F | F | F | F |
| Q15 | F | F | F | F | F | TF |
| Q16 | F | F | F | F | F | F |
| Q17 | F | F | F | F | F | F |
| Q18 | F | F | F | F | F | F |

Ability to explain the questionnaire:

| Questions |  | P1 | P2 | P3 | P4 | P5 | P6 |
| --- | --- | --- | --- | --- | --- | --- | --- |
| Q1 | Poor appetite | Kurang selera makan | Kurang selera makan | Kurang selera makan | Kurang selera | Kurang selera makan | Kurang selera makan |
| Q2 | Not sleeping well | Tidur tidak nyenyak | Tidur tidak nyenyak | Tidur tidak nyenyak | Tidak nyenyak | Tidur tidak nyenyak | Tidur tidak nyenyak |
| Q3 | Irritable, cranky, fussy | Asyik menangis, tidak selesa | Asyik menangis, cepat marah, tidak selesa | Asyik menangis, cepat marah, tidak selesa | Tidak selesa | Emosi tidak stabil | Asyik menangis, cepat marah, tidak selesa |
| Q4 | Feels unwell | Rasa tidak sihat | Rasa tidak sihat | Merengek | Rasa tidak sihat | Tidak selesa | Rasa tidak sihat |
| Q5 | Low energy, tired | Kurang bertenaga, letih | Kurang bertenaga, letih | Kurang bertenaga, letih | Lemah | Kurang bertenaga, letih | Kurang bertenaga, letih |
| Q6 | Not playing well | Tidak bermain seperti biasa | Tidak bermain seperti biasa | Tidak bermain seperti biasa | Tidak aktif | Tidak macam selalu | Tidak bermain seperti biasa |
| Q7 | Crying more  than usual | Menangis lebih daripada biasa | Menangis lebih daripada biasa | Menangis lebih daripada biasa | Menangis lebih daripada biasa | Menangis lebih kerap | Menangis lebih daripada biasa |
| Q8 | Needing extra care | Perlu lebih penjagaan | Perlu lebih penjagaan | Perlu lebih penjagaan | Perlu lebih penjagaan | Lebih perhatian | Perlu lebih penjagaan |
| Q9 | Clinginess | Selalu berpaut | Berpaut | Berpaut | Berpaut | Suka berdukung | Respon ketika didukung |
| Q10 | Headache | (sukar untuk ketahui kerana masih bayi) | (sukar untuk ketahui kerana masih bayi) | (sukar untuk ketahui kerana masih bayi) | Sakit kepala | Sakit kepala | Sakit kepala |
| Q11 | Sore throat | Sakit tekak | (hanya boleh diketahui oleh doktor) | (hanya boleh diketahui oleh doktor) | Sakit tekak | Sakit tekak | Sakit tekak |
| Q12 | Muscles aches  or pains | (susah untuk ketahui kerana masih bayi) | (sukar untuk ketahui kerana masih bayi) | (sukar untuk ketahui kerana masih bayi) | Sakit otot | Sakit otot | Sakit otot |
| Q13 | Fever | Demam | Demam | Demam | Demam | Demam | Demam |
| Q14 | Cough | Batuk | Batuk | Batuk | Batuk | Batuk | Batuk |
| Q15 | Nasal congestion, runny nose | Selsema | Hidung tersumbat, hingus meleleh | Hidung tersumbat, hingus meleleh | Selsema | Selsema | Selsema |
| Q16 | Vomiting | Muntah | Muntah | Muntah | Muntah | Muntah | Muntah |
| Q17 | Not interested in what’s going on | Tidak berminat dengan apa yang berlaku | Tidak berminat dengan apa yang berlaku | Tidak berminat dengan apa yang berlaku | Tidak berminat | Tidak hirau persekitaran | Tidak berminat dengan apa yang berlaku |
| Q18 | Unable to get  out of bed | Lemah | Tidak berupaya melakukan kebanyakan aktiviti harian akibat batuk teruk | (tidak sesuai kerana masih bayi) | Lemah | Tidak berdaya bangun dari katil | Tidak berdaya bangun dari katil |
